# Supplementary figures and images for: A cross-sectional investigation of SARS-CoV-2 seroprevalence and associated risk factors in children and adolescents in the United States
Source: PLoS One. 2021 Nov 8;16(11):e0259823. doi: 10.1371/journal.pone.0259823 (PMC8575286; doi:10.1371/journal.pone.0259823)

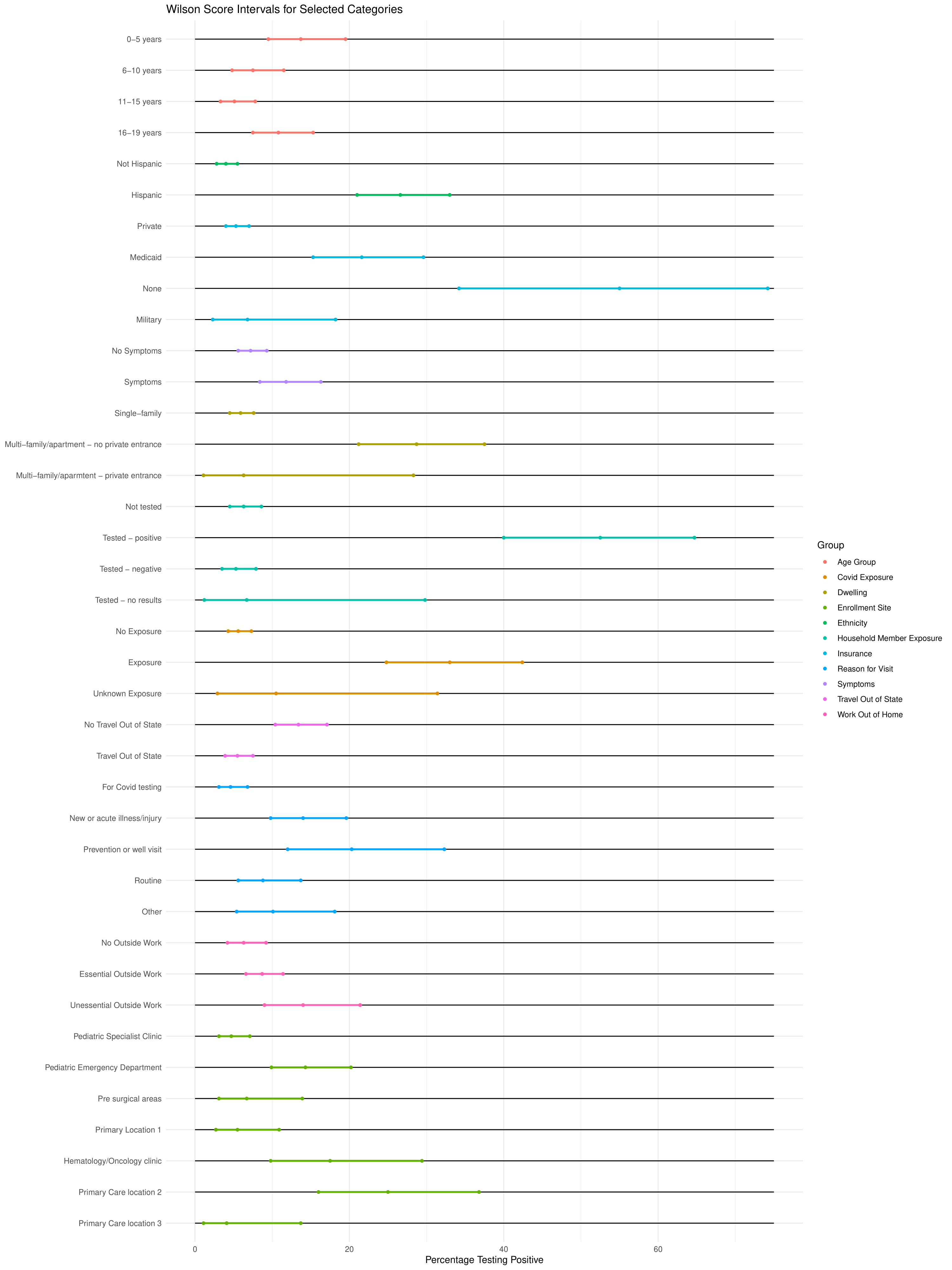

Supplement: S1 Fig — (TIFF) [file pone.0259823.s001.tiff]

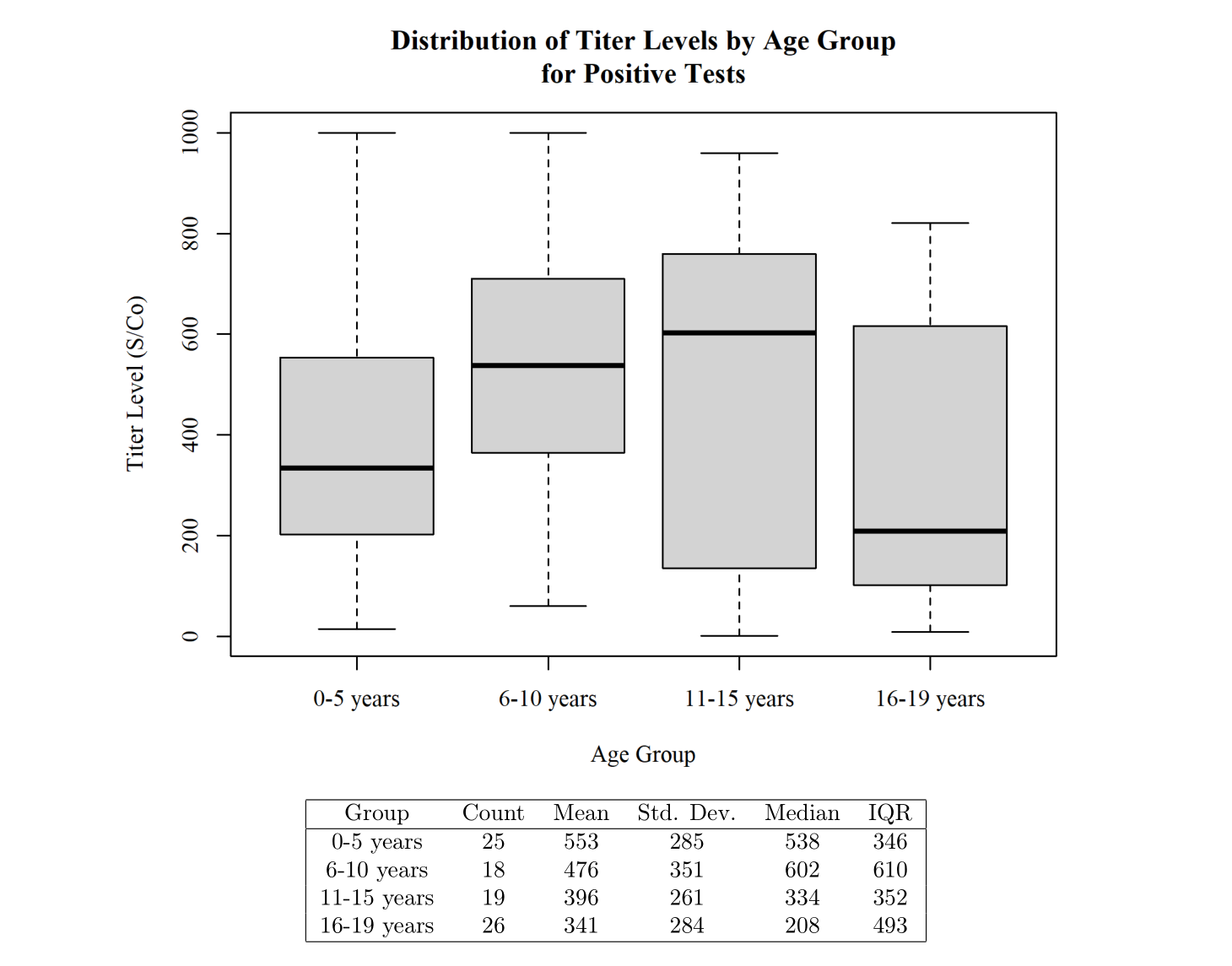

Supplement: S2 Fig — (TIFF) [file pone.0259823.s002.tiff]

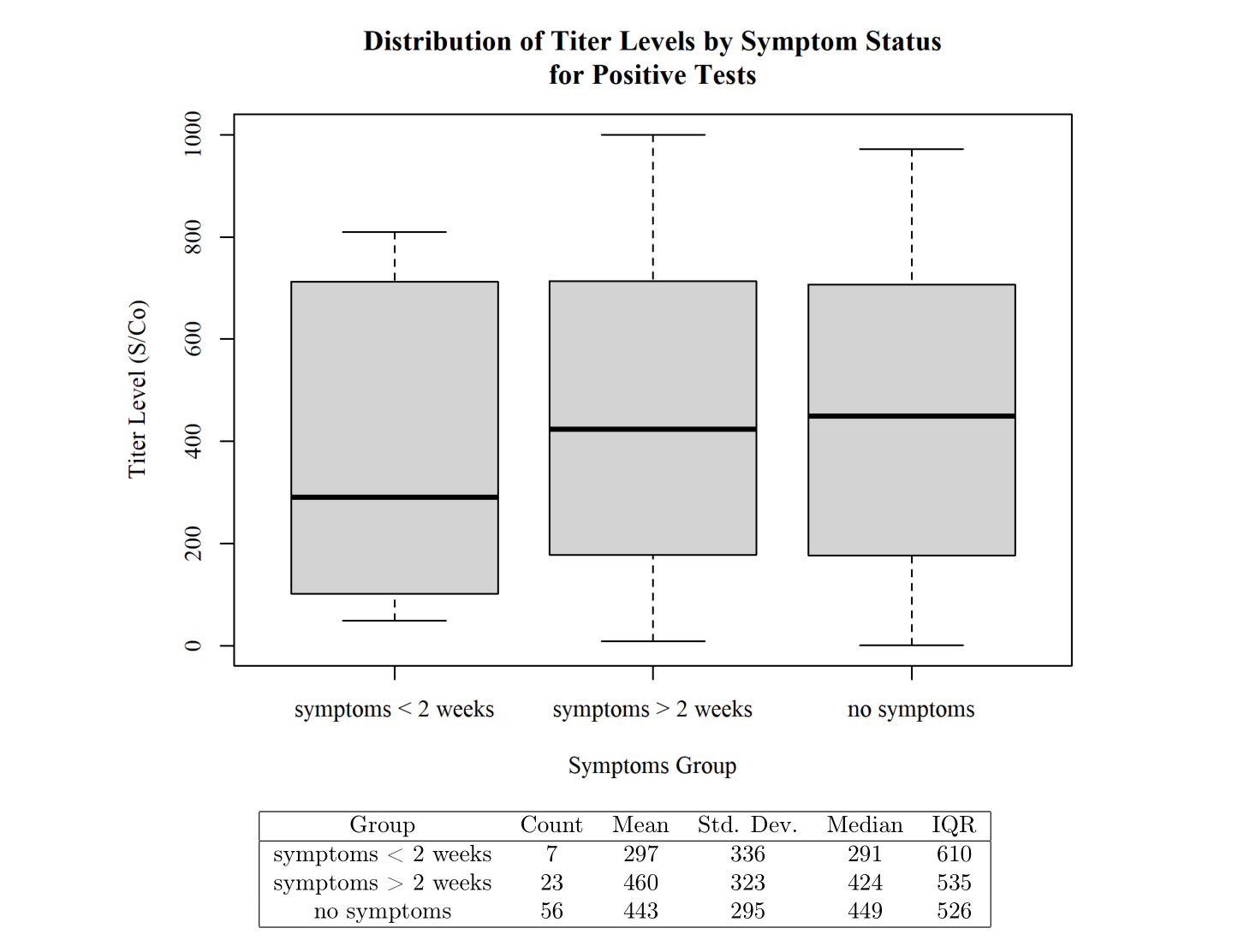

Supplement: S3 Fig — (TIFF) [file pone.0259823.s003.tiff]
