# Supplementary material for: A cross-sectional investigation of SARS-CoV-2 seroprevalence and associated risk factors in children and adolescents in the United States
Source: PLoS One. 2021 Nov 8;16(11):e0259823. doi: 10.1371/journal.pone.0259823 (PMC8575286; doi:10.1371/journal.pone.0259823)
Supplement: S1 Table — (DOCX) [file pone.0259823.s004.docx]

| **Supplemental Table 1: Univariate and Multivariate Logistic Regression Results** | | | | | | | |
| --- | --- | --- | --- | --- | --- | --- | --- |
| **Covariate** | | **Covariate p-value** | | **Level** | **Level p-value** | | |
|  |  | **Univariate** | **Multivariate** |  | **Univariate** | **Multivariate** | |
| Gender | | 0.763 | 0.749 | Male | Baseline | | |
|  |  |  |  | Female | 0.924 | 0.998 | |
|  |  |  |  | Not Disclosed | 0.981 | 0.996 | |
| Age Group | | 0.003 | 0.009 | 0 – 5 years | < 0.001 | 0.534 | |
|  |  |  |  | 6 – 10 years | 0.227 | 0.857 | |
|  |  |  |  | 11 – 15 years | Baseline | | |
|  |  |  |  | 16 – 19 years | 0.009 | 0.534 | |
| Race | | 0.051 | 0.143 | White | Baseline | | |
|  |  |  |  | Black or African American | 0.296 | 0.202 | |
|  |  |  |  | Asian | 0.363 | 0.701 | |
|  |  |  |  | Native Hawaiian or Other Pacific Islander | 0.990 | 0.997 | |
|  |  |  |  | Other | 0.008 | 0.129 | |
|  |  |  |  | Unknown/Declined | 0.984 | 0.996 | |
| Ethnicity | | < 0.001 | < 0.001 | Not Hispanic or Latino | Baseline | | |
|  |  |  |  | Hispanic or Latino | < 0.001 | 0.001 | |
|  |  |  |  | Unknown | 0.985 | 0.998 | |
| Insurance | | < 0.001 | 0.002 | Private | Baseline | | |
|  |  |  |  | Medicaid | < 0.001 | 0.065 | |
|  |  |  |  | Medicare | 0.383 | 0.448 | |
|  |  |  |  | None or uninsured | < 0.001 | < 0.001 | |
|  |  |  |  | Other | 0.075 | 0.126 | |
|  |  |  |  | Don’t know | 0.125 | 0.802 | |
|  |  |  |  | Military | 0.659 | 0.046 | |
| Existing Medical Comorbidities | | 0.739 | 0.554 | No | Baseline | | |
|  |  |  |  | Yes | 0.780 | 0.518 | |
|  |  |  |  | Don’t know | 0.981 | 0.996 | |
| Symptoms | | 0.021 | 0.033 | No | Baseline | | |
|  |  |  |  | Yes | 0.025 | 0.025 | |
|  |  |  |  | Unknown | 0.034 | 0.464 | |
| Dwelling | | < 0.001 | 0.005 | Single-family | Baseline | | |
|  |  |  |  | Multi-family or apartment – no private entrance | < 0.001 | 0.006 | |
|  |  |  |  | Multi-family or apartment– private entrance | 0.951 | 0.734 | |
|  |  |  |  | Other | 0.292 | 0.554 | |
| Household Member Tested for COVID-19 | | < 0.001 | < 0.001 | Not Tested | Baseline | | |
|  |  |  |  | Tested – Positive | < 0.001 | < 0.001 | |
|  |  |  |  | Tested – Negative | 0.531 | 0.097 | |
|  |  |  |  | Tested – No Results | 0.949 | 0.655 | |
|  |  |  |  | Unknown if Tested | 0.986 | 0.997 | |
| Child Exposed to COVID-19 | | < 0.001 | 0.056 | No | Baseline | | |
|  |  |  |  | Yes | < 0.001 | 0.114 | |
|  |  |  |  | Unknown | 0.368 | 0.066 | |
| Traveled Out of State | | < 0.001 | 0.218 | No | Baseline | | |
|  |  |  |  | Yes | < 0.001 | 0.114 | |
|  |  |  |  | Unknown | 0.983 | 0.998 | |
| Traveled Out of Country | | 0.580 | 0.234 | No | Baseline | | |
|  |  |  |  | Yes | 0.565 | 0.076 | |
| Reason for Visit | | < 0.001 | 0.243 | Specifically for COVID antibody testing | Baseline | | |
|  |  |  |  | New or acute illness or injury | < 0.001 | 0.287 | |
|  |  |  |  | Prevention or well visit | < 0.001 | 0.422 | |
|  |  |  |  | Routine visit for a prior condition | 0.034 | 0.343 | |
|  |  |  |  | Other | 0.038 | 0.596 | |
| Work Out of Home | | 0.041 | 0.222 | None | Baseline | | |
|  |  |  |  | Essential | 0.178 | 0.424 | |
|  |  |  |  | Unessential | 0.008 | 0.173 | |
|  |  |  |  | Yes, Not Sure if Essential | 0.105 | 0.657 | |
| Childcare out of Home | | 0.807 | 0.056 | No | Baseline | | |
|  |  |  |  | Yes | 0.807 | 0.052 | |
| Household Size | | 0.239 | 0.439 | --- | 0.230 | 0.283 | |
| Number of Children in Household | | 0.145 | 0.013 | --- | 0.154 | 0.014 | |
| Enrollment Site | < 0.001 | | 0.883 | Pediatric Emergency Department | Baseline | | |
|  |  |  |  | Pre surgical areas | 0.079 | | 0.539 |
|  |  |  |  | Endoscopy suite | 0.985 | | 0.993 |
|  |  |  |  | Pediatric inpatient units | 0.988 | | 0.995 |
|  |  |  |  | Pediatric Intensive Care Unit | 1.000 | | 0.778 |
|  |  |  |  | Pediatric Specialists Clinics | < 0.001 | | 0.879 |
|  |  |  |  | Hematology/Oncology clinic | 0.551 | | 0.378 |
|  |  |  |  | Primary Care location 1 | 0.017 | | 0.899 |
|  |  |  |  | Primary Care location 2 | 0.055 | | 0.543 |
|  |  |  |  | Primary Care location 3 | 0.070 | | 0.726 |
|  |  |  |  | All other Primary Care clinics | 0.985 | | 0.995 |
