# Supplementary material for: A cross-sectional investigation of SARS-CoV-2 seroprevalence and associated risk factors in children and adolescents in the United States
Source: PLoS One. 2021 Nov 8;16(11):e0259823. doi: 10.1371/journal.pone.0259823 (PMC8575286; doi:10.1371/journal.pone.0259823)
Supplement: Attachment — Submitted filename: Response to reviewers.docx [file pone.0259823.s005.docx]

Dear Reviewers and Editorial office,

Thank you for taking the time to review our manuscript and your positive feedback. The answers to your comments are below in bold. We feel these changes made have strengthened the manuscript, thank you.

Yours Sincerely,

Suchi Hourigan and all co-authors

**Response to Reviewers comments**

**Reviewer #1:** The authors performed seroepidemiologic evaluation of SARS-CoV-2 in children in a major metropolitan region of the US. Three FDA approved serology tests were used to assess seroprevalence, Vitros as a primary assay, followed by Orthogonal testing with Siemens and Architect. The observed seroprevalence was higher than what is reported in the literature, I have questions in this regard:
1- Vitros (ortho clinical assay) measures the total antibody to the full spike protein, do you think that the use of Vitros as a primary assay for screening overestimated the seroprevalence in children? as you mentioned in the discussion line 291 “these lower titers levels may represent cross-reactivity to conserved epitopes in benign seasonal human coronavirus” which I agree with. I suggest you cite this paper to support your findings (JCI Insight. 2021;6(4):e144499. [https://doi.org/10.1172/jci. insight.144499](https://doi.org/10.1172/jci.%20insight.144499).).

**Thank you for taking the time to review out manuscript. We are in agreement with your insightful comment. We have expanded line 291 to now read “ Future consideration should be given to redefining the lower limit of detection for full spike protein antibody assays as it is possible that these lower titer levels may represent cross-reactivity to conserved epitopes in benign seasonal human coronaviruses [10, 31], with the Ortho Clinical assay overestimating the seroprevalence in children; however it is not possible to confirm this in our study cohort.” and cited the additional paper you recommended (31).**

Reviewer #2: The manuscript by Suchitra K Hourigan and colleagues, titled “SARS-CoV-2 Seroepidemiology in Children"presents a study and test performance data from SARS-CoV-2 spike protein levels in children and adolescents ≤19 years. The work was well-researched, and the authors mined good techniques to evaluate the performance of the assays in detecting SARS-CoV-2 antibodies. There are minor comment:

1#Since the samples were taken in the period July-Octobar 2020. It would be good to plot this months to see which month was most effected to link this date to occasion holidays or schools.

**Thank you for taking the time to review out manuscript. This is an interesting point which we looked in to further. The positivity rate by month is as follows**

**July: 0% (0/2)**

**August: 8.49% (46/542)**

**September: 10.03% (29/289)**

**October: 6.34% (13/205)**

**There was no statistically significant difference in rates by month and therefore we did not think it was worthwhile having a separate figure for this. However, we have added the following to the text, line 222 “There was no significant difference in positivity rate by month in which the study was conducted.”**

Response to Editorial Office comments:

1. Please ensure that your manuscript meets PLOS ONE's style requirements, including those for file naming. The PLOS ONE style templates can be found at

**DONE**

https://journals.plos.org/plosone/s/file?id=wjVg/PLOSOne_formatting_sample_main_body.pdf and

https://journals.plos.org/plosone/s/file?id=ba62/PLOSOne_formatting_sample_title_authors_affiliations.pdf

2. Please modify the title to ensure that it is meeting PLOS’ guidelines (https://journals.plos.org/plosone/s/submission-guidelines#loc-title). In particular, the title should be "specific, descriptive, concise, and comprehensible to readers outside the field" and in this case it does not meet our requirements in the current form and it is not informative and specific about your study's scope and methodology.

**DONE**

3. Please amend your current ethics statement to address the following concerns: Please explain why written consent was not obtained, how you recorded/documented participant consent, and if the ethics committees/IRBs approved this consent procedure.In addition, please confirm that consent was obtained from parents or guardians of children <18.

**Thank you. To clarify the ethics statement has been changed to:**

**“The Inova Health Systems Human Research Protection Office waived approval as per CFR 45 46.102, as this project was a public health surveillance activity. Verbal informed consent was obtained from parents (for children <18 years) and adolescents ≥18 years and documented by study staff in an electronic database. Assent was additionally obtained for those over 7 years of age. Written consent was not obtained as verbal consent was more feasible and this study was not deemed as research.”**

4. Thank you for stating the following in the Funding Section of your manuscript:

“The Virginia Department of Health provided funding for this study. The Virginia Department of Health, in conjunction with the authors of this manuscript, were involved in study design, the writing of the report and the decision to submit the paper for publication. The Virginia Department of Health were not involved in collection, analysis or interpretation of the data. Research reported in this publication was also supported in part by the National Institute of Child Health and Human Development under Award Number K23HD099240 (Hourigan). Research reported in this publication was also supported in part via a subcontract from the parent award by the National Center for Advancing Translational Sciences of the National Institutes of Health under Award Number UL1TR003015 (Hunter, Sun, Bruce). The content is solely the responsibility of the authors and does not necessarily represent the official views of the National Institutes of Health”

We note that you have provided formation within the Funding. Please note that funding information should not appear in the other areas of your manuscript. We will only publish funding information present in the Funding Statement section of the online submission form.

**Thank you. There is no other funding statement in the manuscript.**

Please remove any funding-related text from the manuscript and let us know how you would like to update your Funding Statement. Currently, your Funding Statement reads as follows:

**The funding statement in the manuscript has now been removed.**

“The Virginia Department of Health provided funding for this study. The Virginia Department of Health, in conjunction with the authors of this manuscript, were involved in study design, the writing of the report and the decision to submit the paper for publication. The Virginia Department of Health were not involved in collection, analysis or interpretation of the data. Research reported in this publication was also supported in part by the National Institute of Child Health and Human Development under Award Number K23HD099240 (Hourigan) . Research reported in this publication was also supported in part via a subcontract from the parent award by the National Center for Advancing Translational Sciences of the National Institutes of Health under Award Number UL1TR003015 (Hunter, Sun, Bruce). The content is solely the responsibility of the authors and does not necessarily represent the official views of the National Institutes of Health. The funders had no role in study design, data collection and analysis, decision to publish, or preparation of the manuscript.”

Please include your amended statements within your cover letter; we will change the online submission form on your behalf.

**Please used the following amended statement:**

**“The Virginia Department of Health provided funding for this study. The Virginia Department of Health, in conjunction with the authors of this manuscript, were involved in study design, the writing of the report and the decision to submit the paper for publication. The Virginia Department of Health were not involved in collection, analysis or interpretation of the data. Research reported in this publication was also supported in part by the National Institute of Child Health and Human Development under Award Number K23HD099240 (Hourigan) and by the Intramural Research Program of the National Institute of Allergy and Infectious Diseases (Hourigan), of the National Institutes of Health. Research reported in this publication was also supported in part via a subcontract from the parent award by the National Center for Advancing Translational Sciences of the National Institutes of Health under Award Number UL1TR003015 (Hunter, Sun, Bruce). The content is solely the responsibility of the authors and does not necessarily represent the official views of the National Institutes of Health. The funders had no role in study design, data collection and analysis, decision to publish, or preparation of the manuscript.”**

5. Please amend either the title on the online submission form (via Edit Submission) or the title in the manuscript so that they are identical.

**DONE**

6.Please review your reference list to ensure that it is complete and correct. If you have cited papers that have been retracted, please include the rationale for doing so in the manuscript text, or remove these references and replace them with relevant current references. Any changes to the reference list should be mentioned in the rebuttal letter that accompanies your revised manuscript. If you need to cite a retracted article, indicate the article’s retracted status in the References list and also include a citation and full reference for the retraction notice.

**DONE. One additional reference (31) added as per reviewer’s suggestion.**
